# From Prediction to Creation: Generative Plant Design

**DOI:** 10.3390/plants15131967

**Published:** 2026-06-26

**Authors:** Juan Ma, Yanzhao Wang, Jianshuang Qi, Zeqiang Cheng

**Affiliations:** Institute of Cereal Crops, Henan Academy of Agricultural Sciences, Zhengzhou 450002, China; yz9839@126.com (Y.W.); qijianshuang@126.com (J.Q.)

**Keywords:** generative plant design, generative AI, Design–Build–Test–Learn cycle, latent space navigation, digital twins

## Abstract

Recent advances in generative modeling have shifted plant breeding from predictive selection to de novo generative design. This review outlines generative methods for navigating the design space and introduces the latent space as a continuous, designable representation that enables a transition from static plant design to dynamic adaptive response programs. We then categorize navigation of the latent space into three strategies: exploration through unconditional generation, guidance through conditional generation, and optimization through feedback loops. We propose a dual-loop generative artificial intelligence-enhanced Design–Build–Test–Learn framework for accelerated plant design. The inner computational loop performs Design–Predict–Optimize guided by causal constraints and virtual evaluators, while the outer experimental loop (Build–Test–Learn) validates elite designs through digital twins and field trials to bridge the reality gap. A proof-of-concept simulation for drought-tolerance design demonstrates the framework’s dual-loop logic and quantitative performance. We further identify five hierarchical challenges that hinder real-world application: the pitfall of continuity assumption, multi-modal data fusion, causal identifiability, and trustworthy evaluation, as well as pleiotropy and genetic load. Finally, we discuss limitations and risks across data, model, regulatory, and interpretability dimensions and highlight critical open questions for realizing dynamic, adaptive, and climate-resilient breeding. This review provides a biology-grounded, systematic framework for next-generation intelligent plant improvement.

## 1. Introduction

Global agriculture stands at a critical crossroad. To meet the food demands of a growing population amidst extreme climate change and diminishing arable land, crop productivity must increase at an unprecedented pace. The transition from Breeding 1.0 to 4.0 has been described as moving from traditional or incidental selection to hybrid, molecular or genomic, and precision or rationally designed breeding [1,2]. Although this transition has greatly increased crop productivity, breeding has remained the screening and recombination of existing genetic resources. Faced with future environmental volatility and increasing resource constraints, this paradigm depending on standing variation is approaching the limits of its effectiveness. This has prompted the recent proposal of Breeding 5.0, or generative breeding [2], a shift that aims to move beyond selection and toward the de novo creation of plant ideotypes.

Realizing this vision will require artificial intelligence (AI). In fact, AI encompasses distinct paradigms with fundamentally different generative and analytical capabilities. Two classes in particular, predictive (discriminative) AI and generative AI, play distinct roles in breeding innovation yet are frequently conflated. Prediction AI learns input–output mappings, ranks existing genetic variants, and identifies optimal combinations within standing genetic variation. By facilitating early selection of superior individuals, the approach has dramatically accelerated genetic gains in plant breeding [3]. However, it often fails when facing samples beyond the training distribution, leading to significant performance degradation [4]. Generative AI, by contrast, is about generation based on learned data distributions. By learning the high-dimensional probability distribution of structural designs, it performs interpolation, traversal, and optimization within a continuous latent representation space [5]. This latent space acts as a navigable framework in which discrete biological sequences are embedded into continuous coordinates, enabling targeted exploration beyond existing variation. A third paradigm, mechanistic modeling, complements these AI approaches by providing biophysical constraints. Driven by biophysical and physiological principles such as functional-structural plant models [6], mechanistic models describe system behavior through equations. Mechanistic models, such as the STEMMUS-SCOPE model used to construct a soil–plant digital twin (DT) [7], can serve as tools for DTs but are not inherently design-oriented.

Generative models, mainly including generative adversarial networks (GANs), variational autoencoders (VAEs), diffusion models, and autoregressive models, have demonstrated transformative potential across biology [8]. These methodological advances lay a foundation that can be directly extended to plant design applications. In plant, generative models have already seen early applications. At the phenotypic level, such models have been applied to synthesize plant imagery, generate semantic annotations, and quantify stress responses [9,10,11]. At the genotypic level, PlantGFM has achieved the generation of protein-coding sequences with experimental validation of transcriptional de novo activity and protein expression in *Nicotiana benthamiana* [12]. These examples represent a shift from passive prediction toward active design in plant improvement.

Several recent reviews have addressed AI applications in plant breeding and molecular design, but none have proposed a unified generative design framework that explicitly separates computational optimization from experimental validation. Xie et al. [13] proposed a data-driven framework for wheat breeding that integrates multi-omics data, knowledge graphs, and AI to enable predictive decision-making under the Breeding 5.0 paradigm. Fu et al. [14] proposed that AI-driven protein engineering, integrating structure prediction, sequence generation, and de novo design, offers a new paradigm for precise plant trait design, with applications spanning disease resistance to environmental sensing, while Yang et al. [8] offered a broad overview of generative AI for general biomolecule design within and beyond the central dogma of molecular biology, and Zhang et al. [15] explored intelligent design in seed science and proposed an “AI for Science” paradigm that integrates high-throughput phenotyping, DT systems, and genome editing into a closed-loop framework for seed innovation. Although these reviews share a common focus on AI in plant science, they do not integrate DTs as hard filters to bridge the computational-to-physical gap. Here, we provide a systematic review of generative AI for plant design and propose a dual-loop generative AI-enhanced Design–Build–Test–Learn (DBTL) framework that addresses these gaps.

The objective of this review is threefold. First, we explore the latent space as a designable representation for plant genetic architectures and survey three navigation strategies, namely unconditional, conditional, and optimization-guided generation. Second, we propose a generative AI-enhanced DBTL cycle that unifies latent space navigation with generative design, incorporating causal constraints, virtual evaluators, and DTs as hard filters. A proof-of-concept simulation applying the framework to drought-tolerance design is presented to demonstrate its operational logic and quantify framework performance. Third, we analyze key challenges and limitations that hinder real-world deployment, and conclude with open questions for adaptive, climate-resilient plant systems.

The review is organized as follows. Section 2 provides a self-contained background on key AI concepts, including machine learning (ML), neural networks, deep learning, predictive AI, generative AI, mechanistic models, and DTs, tailored for plant science readers. Section 3 surveys generative methods for navigating plant design spaces and categorizes navigation strategies. Section 4 presents the proposed dual-loop generative AI-enhanced DBTL framework and a proof-of-concept simulation. Section 5 analyzes five key challenges for real-world deployment, and Section 6 examines limitations and risks, concluding with open questions.

## 2. Background: Key AI Concepts for Plant Scientists

AI is a broad field that encompasses computational approaches to tasks that typically require human intelligence. ML is a core subset of AI in which models learn patterns from data without explicit programming. Within ML, neural networks are computational architectures inspired by biological neurons, consisting of interconnected layers that transform input data through weighted connections. Deep learning refers to neural networks with many layers, which can automatically learn hierarchical representations from raw data, from simple features to complex patterns, making them particularly effective for high-dimensional biological data such as genomes or transcriptomes [16]. These methods form the technical foundation for most AI applications in plant science.

Building on the predictive and generative AI paradigms introduced in Section 1, we now examine their distinctions alongside mechanistic modeling and DTs. These four approaches play complementary roles in plant design. While predictive and generative AI offer data-driven capabilities, mechanistic models provide biophysical constraints, and DTs integrate these components to mirror real-world system behavior. The key differences among these approaches, including their objectives, strengths, limitations, and data requirements, are summarized in Table 1, which also maps each approach to its specific role within the dual-loop framework proposed in Section 4.

## 3. Generative Design and Strategic Navigation of the Plant Latent Space

### 3.1. Generative Methods for Navigating the Design Space

**Figure 1:**
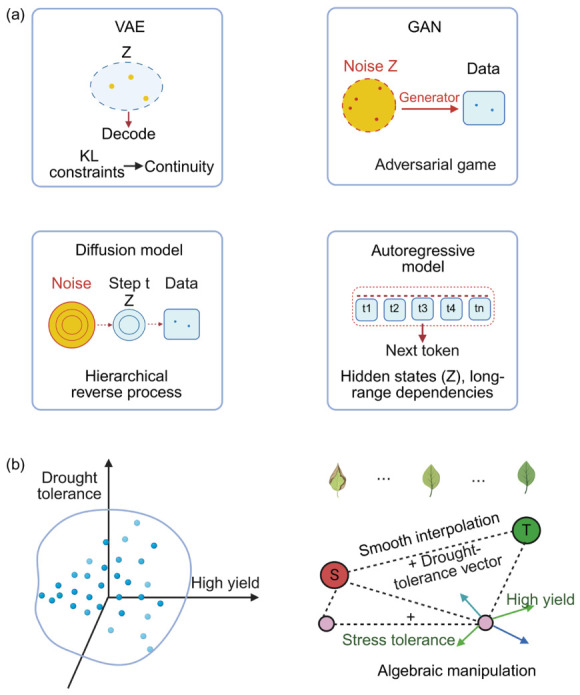
Generative model architectures and latent space manipulation for plant design. (**a**) Latent space characteristics of four generative models. Variational autoencoders (VAEs) impose a continuous, structured latent space through Kullback–Leibler divergence penalty. Generative adversarial networks (GANs) derive a latent space from input noise through an adversarial game. Diffusion models learn a hierarchical latent structure by inverting a gradual noising process, with latent space implicitly defined across time steps. Autoregressive models frame generation as sequential token prediction, capturing long-range dependencies but lacking global continuity. Z denotes the latent space. (**b**) Latent space manipulation for plant design. **Left**: Plant genotypes are embedded in a high-dimensional latent space, with axes corresponding to key agronomic traits including drought tolerance and yield potential. **Right**: Operations in this latent space. Smooth interpolation (dashed line) generates continuous phenotypic transitions from drought-sensitive to drought-tolerant phenotypes (leaf icons) along the drought-tolerance semantic vector. Algebraic manipulation (colored arrows) enables multi-trait optimization: green arrows represent trait vectors for high yield and stress tolerance, whose vector addition yields latent representations corresponding to genotypes with synergistic traits. Additional colored arrows denote manipulation directions for other traits. Created in BioRender. Ma, J. (2026) https://BioRender.com/8sugrnp, accessed on 24 June 2026.

Generative architectures differ fundamentally in how they learn and navigate latent representations (Figure 1a). VAEs explicitly construct a structured latent space by enforcing continuity through a Kullback–Leibler divergence penalty [17]. GANs learn the data distribution through an implicit adversarial game [18]. Here, the latent space is defined by the input noise distribution fed into the generator. Diffusion models learn to reverse a gradual denoising process [19], thereby forming a temporally hierarchical latent structure. Autoregressive models such as Transformers treat generation as sequential token prediction; their hidden states capture long-range dependencies [20], though the representation is not globally continuous.

### 3.2. Latent Space: A Continuous and Designable Representation

Applying generative AI for plant breeding requires defining its operational domain: the biological design space. This concept refers to the vast, high-dimensional, discrete space of all possible genetic sequences. Directly searching within raw sequence space is intractable due to the curse of dimensionality and the absence of navigable gradients. Generative models learn a non-linear mapping from the discrete, symbolic biological space to a continuous, structured latent space. While the latent space encodes genomic features from genetic sequences, its geometry can be organized such that different directions correspond to specific phenotypic traits. This latent representation offers three key advantages (Figure 1b). First, continuity makes the space navigable: proximate points correspond to functionally similar entities, enabling smooth interpolation between discrete states. The continuity of latent space is well-established in image generation through smooth interpolation [21]. This property extends to biological sequences, as protein language models embed discrete amino acid sequences into a continuous vector space [22].

Second, the latent space embeds semantic directions aligned with agronomic traits. If the latent space contains a vector that points from sensitive to tolerant phenotypes, adding this vector to a candidate’s latent representation should produce a new representation with enhanced tolerance (Figure 1b). Such vector-based manipulations have been validated in plant morphological features, where latent vectors controlling size, shape, and ornamentation of pollen grains have been successfully manipulated using StyleGAN [23]. By analogy, a transferable rule for generative plant design emerges: adding a “high yield” vector learned from one genotype to the latent representation of a stress-tolerant genotype should enable the rational combination of complex agronomic traits (Figure 1b).

Third, algebraic operations in latent space correspond to biologically meaningful manipulations. This principle was first popularized in fields such as computer vision through vector arithmetic of concepts [21], and subsequently validated in single-cell genomics through perturbation vectors [24] and in plant morphology by manipulating disentangled latent vectors that separately encode base shape and 3D deformations in leaf architecture [11]. These cross-domain validations suggest that latent spaces can serve as a unified computational interface, where semantic vectors (e.g., “higher yield” or “enhanced drought tolerance”) become tangible design variables.

### 3.3. From Static Design to Dynamic Response Programs

The latent space paradigm described above establishes a unified framework for design, allowing discrete genetic elements to be embedded in a continuous space where functional traits can be systematically manipulated. In Breeding 5.0, however, the design objective evolves from a static ideotype to a dynamic response program that senses and adapts to environmental fluctuations. This requires that the latent representation encode not just snapshots of phenotypic states, but temporal trajectories across changing environments. Learning latent dynamics through neural ordinary differential equations provides a direct strategy to achieve this goal. For instance, scNODE integrates VAEs and neural ordinary differential equations with dynamic regularization to learn temporal manifolds from single-cell data [25]. Although these methods focus on inferring dynamics from time-series data, they show how to learn continuous trajectories in latent space, which can be used for generative plant design to synthesize genetic architectures that respond to environmental fluctuations.

### 3.4. Strategic Navigation of the Latent Space

#### 3.4.1. Exploration Through Unconditional Generation

**Figure 2:**
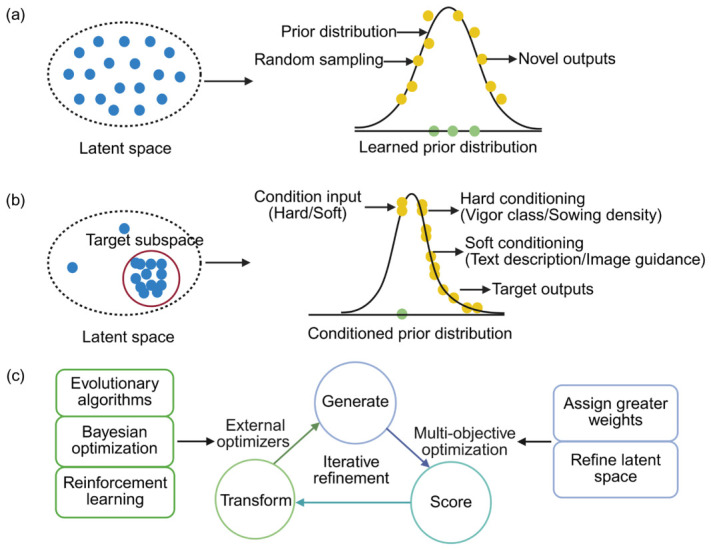
Three strategies for navigating the latent space in generative plant design. (**a**) Exploration through unconditional generation. Unconditional generation relies on random sampling from the learned prior distribution of the latent space, enabling exploration of novel phenotypic variants. (**b**) Guidance through conditional generation. Conditional generation implements targeted sampling within a constrained latent subspace, guided by either hard conditions (discrete attributes such as vigor class and sowing density) or soft conditions (cross-modal signals including text descriptions and image guidance), to produce design-specific target outputs. (**c**) Optimization through feedback loops. The “generate–score–transform” pipeline [29] integrates external optimizers and multi-objective scoring to iteratively refine the latent space and prioritize high-performing designs. Created in BioRender. Ma, J. (2026) https://BioRender.com/3mm9pzf, accessed on 24 June 2026.

Generative models can navigate the latent space toward desired properties through distinct strategies, starting with unconditional exploration. Unconditional generation explores novelty by randomly sampling from the learned prior distribution (Figure 2a). This strategy has primarily been used to augment plant phenotypic data, such as image augmentation [26,27], rather than to directly generate genotypic designs such as promoters, enhancers, or gene circuits. The lag may be partly attributable to the polyploidy and complex repeat structures of plant genomes, which challenge current model architectures [28]. Recent advances, such as PlantGFM [12], have begun to bridge this gap by demonstrating de novo generation of full-length gene sequences, yet the broader field remains focused on phenotypic applications. Regardless of domain, unconditional generation has an intrinsic limitation: it performs well in interpolation within its training distribution but cannot reliably and controllably target regions with specific design properties.

#### 3.4.2. Guidance Through Conditional Generation

Conditional generation uses target conditions, such as desired traits or environmental contexts, to guide generation toward specific subspaces (Figure 2b), converting undirected exploration into targeted design. In plant phenotyping, hard conditioning relies on explicit, predefined inputs. For instance, conditional GANs guided by vigor class labels generate trait-specific plant images [30]; multi-conditional Wasserstein GANs incorporating sowing density, cultivar, and time as conditions simulate dynamic growth trajectories [31]. Soft conditioning operates through implicit or cross-modal constraints. Text-guided diffusion models generate plant images from natural language descriptions [32]. Unsupervised image-to-image translation models (e.g., CycleGAN) provide another form of soft conditioning, generating synthetic training data for cross-species stomatal segmentation and reducing dependency on manual annotation [33]. Besides static image translation, diffusion-based frameworks such as Agricrafter generate full-growth-cycle crop videos from a single seedling image conditioned on agronomic parameters [34]. Together, these hard and soft conditioning approaches remain predominantly applied to phenotypic generation and analysis, rather than the rational design of genotypes. Recently, conditional generation has begun to extend from phenotypic to genotypic design. Xiang et al. [35] proposed TargetGAN, a framework that integrates a GAN with a pre-trained activity predictor. By backpropagating the difference between predicted and user-defined target activity through the latent space, TargetGAN enables the *de novo* design of plant core promoters with specified transcriptional activity.

#### 3.4.3. Optimization Through Feedback Loops

Optimizing multiple traits often requires iterative refinement, an approach known as optimization-guided generation (Figure 2c). For example, in multi-objective optimization, ranking molecules by Pareto optimality and assigning greater training weights to higher-ranked candidates allows refinement of the latent space in junction-tree VAEs without ad hoc scalarization [36]. The evedesign framework achieves seamless integration of heterogeneous models through standardized “generate–score–transform” operations, offering composable infrastructure for multi-objective optimization and closed-loop laboratory design [29]. The efficiency of the generate–score–transform loop can be further enhanced by incorporating external optimization algorithms (e.g., evolutionary algorithms, Bayesian optimization, reinforcement learning). For instance, evolutionary algorithms have been successfully applied to optimize plant tissue culture media within defined design spaces [37]. In plants, however, existing applications of multi-objective optimization within feedback loops remain limited to coupling with process-based mechanistic models or deep learning-based predictors [38,39]. This gap underscores the need to extend optimization-guided generation frameworks toward rational design of plant phenotypes and genotypes.

## 4. A Generative Artificial Intelligence-Enhanced Design–Build–Test–Learn Framework

### 4.1. Overview of the Proposed Dual-Loop Framework

**Figure 3:**
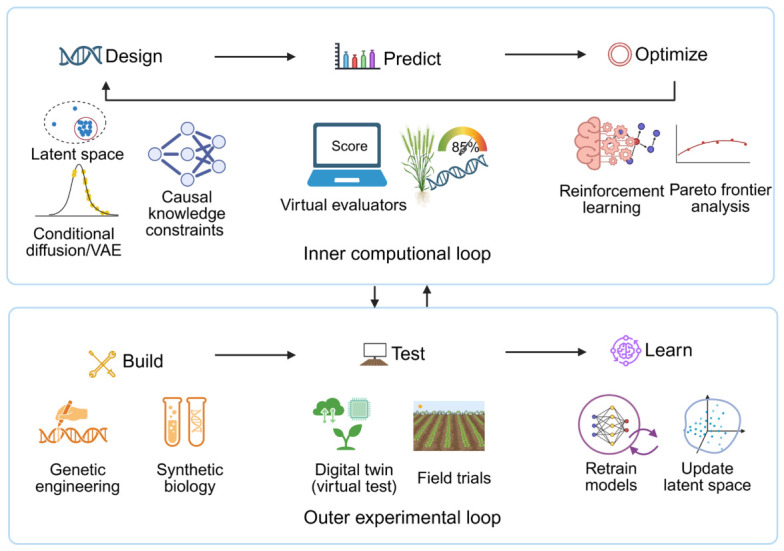
A dual-loop generative AI-enhanced DBTL framework for plant design. The framework integrates an inner computational loop (Design–Predict–Optimize) and an outer experimental loop (Build–Test–Learn) to accelerate generative plant design, incorporating causal constraints, virtual evaluators, and digital twins as hard filters. The inner loop performs design, prediction, and optimization iteratively. Design generates biologically plausible genetic variants using conditional generative models, constrained by causal knowledge derived from multi-omics networks. Prediction employs virtual evaluators for high-throughput phenotypic screening of candidate designs. Optimization applies reinforcement learning and Pareto analysis to prioritize high-performing candidates. In the outer loop, the Build phase engineers the optimized candidates genetically. Subsequently, the Test phase subjects these engineered variants to digital twin filtering and field trials. Finally, the Learn phase performs model calibration and latent space updates. Experimental data from the Learn module feeds back to the inner loop, closing the full cycle and continuously improving framework performance. Created in BioRender. Ma, J. (2026) https://BioRender.com/l1o86eb, accessed on 24 June 2026.

To implement the generative design strategies outlined above, we propose a framework inspired by the DBTL cycle of synthetic biology, tailored to the unique constraints of plant breeding. The conventional DBTL framework’s single-loop architecture limits rapid iteration for generative design. Our framework overcomes this limitation by introducing a dual-loop architecture: an inner computational loop following a Design–Predict–Optimize cycle, and an outer experimental loop following a Build–Test–Learn cycle (Figure 3).

Within the inner loop, a generative model, such as a conditional diffusion model or a VAE, operates in a learned latent space. It generates candidate genetic configurations that align with target traits defined by the breeders. These candidates are generated under causal constraints, rapidly evaluated by predictive models, and refined via closed-loop optimization. For multi-trait breeding objectives (e.g., yield and drought tolerance), the generator can be conditioned on multiple targets simultaneously to navigate trade-offs between competing traits. In the outer loop, candidates are first built through genetic engineering and synthetic biology methods. They are then screened via DT filtering (as a hard filter) and field trials. Finally, the results retrain generative models and update the latent space. We term this system the generative AI-enhanced DBTL framework, representing a paradigm shift in plant breeding from empirical screening toward a systematic engineering discipline.

Compared with existing computational frameworks that employ generative models for specific optimization tasks, such as the dual-population multi-objective evolutionary algorithm for protein-peptide docking [40], our framework operates at a broader scope of abstraction. Rather than addressing a single molecular optimization problem, it is designed to encompass the entire plant design cycle, from in silico candidate generation to field validation, with explicit experimental feedback closing the loop.

### 4.2. Causal Constraints in the Design Step

During the design phase of the inner loop, generative models explore genetic configurations and their regulatory circuits within latent space. However, statistically plausible designs are not necessarily biologically feasible. Causal knowledge extracted from multi-omics data provides a rational way to restrict the generative space [41]. Such constraints, often inferred from perturbation experiments or time-series data, are inherently statistical. Therefore, they can be embedded into generative models as inductive biases, regularization terms, or structural priors, steering generation toward biologically viable candidates while maintaining exploration capacity. The feasibility of embedding causal constraints in generative models has been demonstrated in plants. For instance, GeneSys integrates lineage blueprints as developmental constraints to simulate single-cell transcriptomic trajectories, supporting causal gene prioritization and dynamic network inference in Arabidopsis root development [42].

### 4.3. Virtual Evaluators in the Predict Step Accelerate Inner Loop Iteration

Candidate designs generated by models require rapid evaluation to guide subsequent iterations. In the inner loop, the Predict step employs predictive models as virtual evaluators, using computational surrogates to estimate candidate performance without physical experimentation. Predictor-guided generation has already been realized across diverse biological systems. For example, from bacteria to plants, predictors including Enformer and Borzoi have been combined with generative models (e.g., Evo 2) to guide sequence generation toward desired functional outputs such as chromatin accessibility, providing reliable feedback signals for generative design [43].

### 4.4. Generators Update in the Optimize Step

After each generation round, all candidates are scored by the virtual evaluators. The generator is subsequently updated to favor candidates with higher predicted performance for target traits. This update can be implemented through reinforcement learning, where the predictor scores act as rewards, or through gradient-based optimization, which treats the predictor as a differentiable objective. In multi-trait optimization, Pareto frontier analysis can be used to identify non-dominated candidates that achieve optimal phenotypic trade-offs. These high-ranking candidates are assigned greater weight in updating the generator, iteratively refining the latent space toward regions that balance multiple breeding objectives. Pareto-based strategies have been successfully applied for multi-objective latent space optimization in generative molecular and protein design [36,44].

### 4.5. Digital Twins as Virtual Validation in the Test Phase

Elite candidate designs obtained from inner loop convergence enter the outer loop for experimental validation. However, not all designs that perform well in silico necessarily exhibit favorable performance in real-world environments. DTs provide a systematic approach to bridging this reality gap. They are digital equivalents of real-world objects that mirror their states and behavior in virtual space and stay synchronized through real-time data exchange [45]. In the proposed framework, DTs operate in the Test phase of the outer loop, after candidates have been physically constructed through genetic engineering. They simulate whole-plant biophysical and physiological processes under realistic field conditions using mechanistic models [46]. These mechanistic models impose hard biophysical constraints including water balance, carbon allocation, and phenological/developmental timing that any biologically realistic plant phenotype must satisfy [47,48]. By serving as a hard filter, DTs eliminate designs that violate these thresholds before they proceed to costly field trials. Consequently, only feasible designs reach physical validation, allowing the outer loop to generate high-quality experimental data. In the subsequent Learn phase, relevant data retrains inner-loop generative models and optimizes latent space, forming a closed prediction-validation feedback loop, as exemplified by automated biofoundry systems [49] and standardized feedback reintegration strategies [50].

The high computational cost of DTs limits their direct use as rapid evaluators in the inner loop. Surrogate models offer computationally efficient alternatives to high-cost simulations [51]. Neural surrogates can be pre-trained to represent the input–output behavior of DTs, enabling high-throughput evaluation while maintaining physical consistency. Such surrogates achieve orders-of-magnitude faster inference with comparable accuracy and strong generalization [52].

### 4.6. Proof-of-Concept Simulation for Drought Tolerance Design

To demonstrate the operational logic of the proposed dual-loop framework, we implemented a proof-of-concept simulation applying the framework to a synthetic drought-tolerance trait in a simplified maize genome. The simulation comprised four outer-loop iterations, each integrating a full inner-loop Design–Predict–Optimize cycle followed by outer-loop Build–Test–Learn validation. The ground-truth biological function was defined as a rugged fitness landscape with additive effects from five loci, pairwise epistatic interactions, and a penalty for extreme allele combinations. The DT was initialized as a simplified linear model and updated after each outer loop using field validation data. The generative model was implemented as a flexible sampler with exploration–exploitation dynamics; it generated 50 novel genotypes per iteration, the DT screened them, and the top five were passed to field validation as a hard filter.

**Figure 4:**
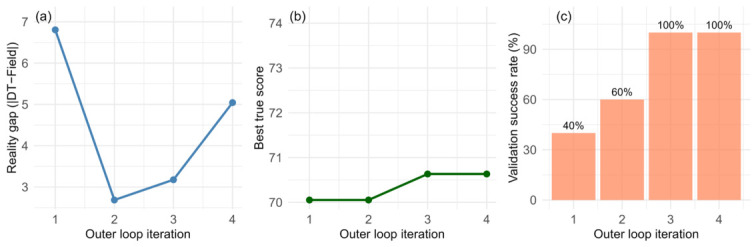
Dual-loop simulation results across four outer-loop iterations. (**a**) Reality gap reduction. (**b**) Best true drought tolerance score improvement. (**c**) Validation success rate increase.

The reality gap decreased from 6.81 in the first iteration to 2.69 in the second (60.5% reduction) (Figure 4), demonstrating that the Learn phase recalibrates the DT. It partially rebounded to 3.18 and 5.04 in subsequent iterations, reflecting that the generative model shifts from exploration to exploitation and samples regions where the DT has limited training data. The validation success rate increased from 40% to 100% by the third iteration, and the best-performing genotype (70.6/100) was identified within three iterations. These results show that the dual-loop framework is not merely conceptual: the inner loop narrows the candidate pool from 50 to 5 per iteration, the outer loop generates field data that systematically shrink the reality gap, and the Learn phase closes the cycle. We emphasize that this is a proof-of-concept demonstration using synthetic data; extension to real genomic data is a direction for future work. The full R code is provided as Supplementary Code S1.

While the proof-of-concept simulation illustrates the operational logic of the dual-loop framework, several implementation requirements must be considered for practical deployment. First, learning meaningful latent spaces for complex polyploid genomes typically requires large-scale, high-quality datasets, which are not always available for many plants. Second, computational costs for training generative models at genome scale are substantial, though transfer learning and foundation models may reduce this burden. Third, field trials are expensive and time-consuming, underscoring the value of DTs as hard filters. Fourth, practical feasibility depends on seamless integration with existing breeding pipelines, requiring user-friendly interfaces and interpretable outputs for breeders.

## 5. Closing the Reality Gap: Challenges and Solutions

### 5.1. Foundational Constraint: The Pitfall of Continuity Assumption

The dual-loop framework faces several challenges when deployed under real-world field conditions. Generative models rely on a continuity assumption that is often inconsistent with the discrete, nonconvex nature of biological design spaces. Mapping plant designs to a continuous latent space facilitates gradient-based navigation but relies on a questionable premise imported directly from computer vision: that plant design spaces are well-behaved and continuous, such that interpolation between functional genotypes reliably produces functional intermediates. The assumption has been extended to generative plant design without sufficient critical validation. Plant systems violate the continuity assumption in three fundamental ways.

**Figure 5:**
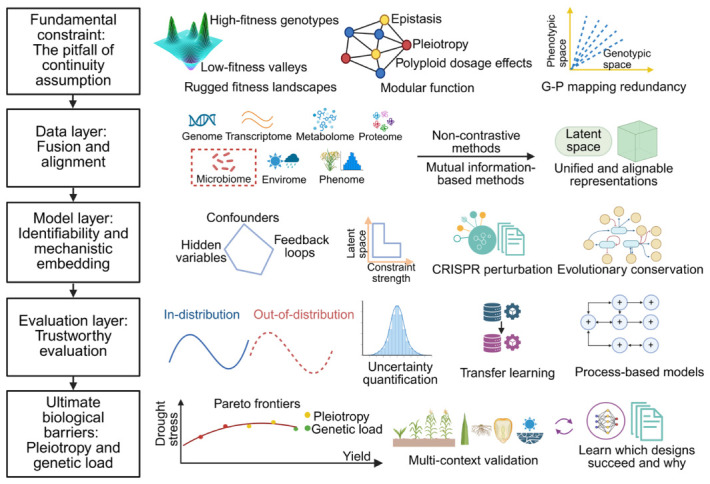
Key computational and biological challenges in generative plant design.

First, plant fitness landscapes are rugged, with viable genotypes clustered on isolated peaks separated by valleys of low fitness (Figure 5). In maize, yield-trait performance landscapes exhibit multiple high-performance peaks and intervening valleys [53]. This ruggedness explains why superior parents do not always produce superior offspring: even high-performing parents can yield hybrids that fall into low-fitness valleys. Second, plant function is modular and non-additive rather than linearly composable. Epistasis, pleiotropy, and polyploid dosage effects break linear assumptions. In particular, maize traits emerge from synergistic gene interactions, not linear aggregation [54]. Third, phenotypic discreteness does not imply simple or low-redundancy genotype-phenotype mapping.

The consequences of the continuity assumption are not merely theoretical. In practice, generative models trained on continuous representations have produced non-viable designs in biological systems, as documented in protein design [55] and plant breeding contexts where continuous genotype representations fail to capture epistatic constraints [56]. These examples underscore that latent space continuity is a computational convenience, not a biological principle, and generative designs that violate this principle are unlikely to survive field validation.

To address this, this review outlines two concrete mitigation strategies for the continuity pitfall. First, hybrid discrete-continuous representations, such as VQ-VAE [57,58], can treat allelic composition as discrete and polygenic dosage as continuous, preserving latent space power while constraining outputs to biologically plausible combinations. This strategy has demonstrated potential as a data augmentation tool; for example, VQ-VAE has been used to generate synthetic hyperspectral data for maize starch prediction, enhancing data diversity and generalization [59]. Second, ruggedness-aware regularization, implemented through topological regularization [60] or input gradient regularization [61], can prevent generative models from smoothing over fitness landscape ruggedness and encourage sensitivity to epistatic interactions. While these approaches have not yet been benchmarked against conventional breeding pipelines, evaluating their performance in plant breeding remains a priority for future work.

The pitfall of continuity assumption. This assumption conflicts with rugged fitness landscapes; modular, non-additive functions shaped by epistasis, pleiotropy, and polyploid dosage effects; and phenotypic discreteness that does not imply simple or low-redundancy genotype-phenotype mapping.

Multi-modal data fusion and alignment. Integrating multi-omics, microbiome, envirome and phenome data relies on principled alignment strategies, including non-contrastive and mutual information-based methods, to construct a unified latent space. The red dashed box highlights the systematic exclusion of microbiomes as a critical gap.

Identifiability and mechanistic embedding. Generative models face causal non-identifiability from confounders, hidden variables, and feedback loops, as well as trade-offs between overly rigid biological constraints that limit novelty and weak constraints that produce unreliable designs. Integrating CRISPR perturbation data and evolutionary conservation can improve identifiability and biological realism.

Trustworthy evaluation. Generative models often fail to generalize to out-of-distribution genetic variants, necessitating robust uncertainty quantification, transfer learning, and integration with process-based mechanistic models to ensure reliable evaluation of novel designs beyond training data.

Pleiotropy and genetic load. Pleiotropy and genetic load impose context-dependent fitness costs that are difficult to predict in silico, making single-trait optimization risky. Pareto-based multi-objective optimization mitigates this by identifying candidates that balance multiple traits. Multi-context validation in the outer loop reveals latent trade-offs, and empirical outcomes feed back to enable iterative learning. Created in BioRender. Ma, J. (2026) https://BioRender.com/f0u6650, accessed on 24 June 2026.

### 5.2. Data Layer: Multi-Modal Data Fusion and Cross-Modal Alignment

Given the rugged nature of the design space, a fundamental challenge is to learn a unified latent space that coherently integrates multi-omics and multi-modal data (Figure 5). Unlike predictive models that tolerate loosely coupled inputs, generative models require tightly aligned cross-modal representations for stable, biologically plausible generation. Traditional fusion approaches cannot ensure this coherence, often yielding misaligned representations. A major source of failure lies in standard contrastive learning, which presents two critical limitations for plant systems. First, it overemphasizes shared information while discarding modality-unique signals essential to genetic improvement [62,63]. Second, most alignment methods fail to capture synergistic relationships, which are especially relevant in plants where genetic synergy drives development. For example, incomplete functional divergence among duplicated *SEPALLATA* floral regulators produces synergistic effects underlying tomato inflorescence development [64], yet such interactions would be overlooked by standard contrastive alignment.

To reduce redundancy while preserving biologically meaningful synergies, standard contrastive learning should be replaced by more principled alignment strategies. Non-contrastive self-supervised methods (e.g., Barlow Twins) learn decorrelated and disentangled representations, mitigating redundancy bias [65]. These principles have recently been adapted to plant phenotyping, where Barlow Twins-based frameworks have been shown to strengthen feature discrimination and boost disease identification performance on limited plant image data [66]. Mutual information maximization frameworks further improve alignment by jointly capturing shared, unique, and synergistic multi-modal interactions [63]. Such mutual information-aware alignment preserves key biological signals and enables generative models to explore novel allele combinations that exploit hidden compensatory regulatory networks. Notably, these strategies, particularly non-contrastive and mutual information-based alignment, can be directly integrated into generative models to construct structured, biologically coherent latent spaces.

A further critical gap is the systematic exclusion of rhizosphere and phyllosphere microbiomes, which strongly modulate fitness, stress resilience, and productivity [67]. While microbial and genomic features have been naively concatenated for prediction [68], such fusion cannot adequately model host–microbe interactions. Next-generation generative models should treat microbial profiles as a core modality equivalent to genomic data. Jointly embedding multi-omics, microbiomes, phenotypes, and environments within a unified, rigorously aligned latent space will enable system-level understanding and generate more robust, field-relevant plant designs.

### 5.3. Model Layer: Causal Identifiability and Mechanistic Embedding

Even with well-aligned multi-modal data, models typically capture statistical correlations rather than true causal relationships. Integrating causal knowledge into generative models faces a core bottleneck in causal identifiability. Causal structures inferred from observational data are often only statistical approximations of real biological mechanisms or entirely spurious correlations driven by unmeasured confounders [69,70], which cannot support reliable generative design. Two key challenges emerge for targeted generative plant breeding. First, causal identifiability cannot be guaranteed. Inferred structures are often non-unique and underdetermined [71], while confounders, hidden variables, and feedback loops collectively impede robust reconstruction [72,73]. A second challenge stems from intrinsic biological constraints that create an inevitable trade-off between realism and novelty. Overly strong constraints restrict the valid design space, while weak or insufficient constraints lead to spurious correlations and biologically implausible outputs (Figure 5).

A resolution is to embed causal validation directly within the generative loop. First, increasingly accessible plant CRISPR perturbation and high-throughput regulatory screens can generate experimentally validated, causal mutation-effect relationships for gene expression [74], which can serve as ground-truth interventional labels to regularize generative models and penalize outputs that violate known regulatory constraints. Second, evolutionary conservation across orthologs provides a robust functional-causal signal to separate conserved regulatory mechanisms from species-specific, non-conserved, or spurious correlations [75]. Beyond validation, plant-specific mechanistic embedding can improve interpretability and biological realism by integrating biophysical, transcriptional control, and breeding principles into model architectures [76]. Soft constraints informed by metabolic network models help narrow the search space while retaining biological plausibility [77]. Nevertheless, dedicated frameworks tailored to complex plant traits and dynamic multi-omics regulation remain largely missing.

### 5.4. Evaluation Layer: Trustworthy Evaluation for Novel Designs

A key challenge in causally grounded generative plant design is the trustworthy evaluation of novel genotypes and their associated phenotypes (Figure 5). Most existing models are trained on historical observational data, which limits their ability to assess truly novel, out-of-distribution designs that generative models aim to produce. Reliance on in-distribution performance can lead to unreliable or misleading assessments, especially when predictions extend beyond the environmental, genetic, or phenotypic scope of existing datasets. In plant phenotyping, Andvaag et al. [78] showed that generalization to unobserved conditions depends more on training set diversity than data volume, which points to a mismatch between current evaluation practices and the needs of generative breeding.

Addressing the out-of-distribution challenge requires a range of uncertainty quantification strategies, including conventional machine learning estimators and deep learning-based approaches. In remote sensing, conventional methods have been rigorously validated for hyperspectral vegetation trait retrieval. García-Soria et al. [79] systematically evaluated these approaches and found that each method provides reliable, physically interpretable uncertainty estimates. Besides uncertainty-aware inference, transfer learning provides an effective strategy to adapt prediction models to unseen environmental and spectral domains [4]. Alternatively, rather than relying on statistical relationships, process-based models grounded in crop physiological and physical mechanisms support mechanistic and causal evaluations instead of only statistical correlations [80].

To make evaluation actionable for generative plant design, we organize it across four dimensions: sample quality and diversity (measured by distributional fidelity and pairwise distance in genotype space), biological validity (whether generated genotypes satisfy known biological constraints, such as epistatic relationships), predictive consistency (whether DT predictions correlate with field outcomes), and breeding-relevant outcomes (field validation success rates and realized genetic gain). No single metric suffices. Trustworthy evaluation requires a combination of statistical, biological, predictive, and agronomic criteria to flag unreliable candidates before costly field validation.

### 5.5. Ultimate Biological Barriers: Pleiotropy and Hidden Genetic Load

After addressing data alignment, causal identifiability, and trustworthy evaluation, a biological reality remains: promising designs may fail in practice due to pleiotropic trade-offs and hidden genetic load that are difficult to anticipate in silico (Figure 5). Generative models optimized for a single trait risk producing designs that are statistically plausible but biologically inviable. For example, drought-tolerant designs that appear optimal in silico may impose yield penalties under non-stress conditions or suffer unexpected fitness costs under field drought stress, failing to deliver their predicted performance in practice. Empirical evidence illustrates the reality of pleiotropic trade-offs. In rice, multi-trait genome-wide association studies identified 44 pleiotropic QTLs affecting yield and yield-related traits, of which 29 were not detected by single-trait methods [81]. These findings underscore that single-trait optimization in generative design may overlook hidden trade-offs that only manifest under field conditions, highlighting the need for multi-objective approaches that account for pleiotropic constraints.

To mitigate these challenges, multi-objective optimization with Pareto-based reward signals offers a practical approach within the generative loop. Instead of optimizing for a single target, the generative model should be conditioned on multiple traits simultaneously, using non-dominated sorting and Pareto-optimal front analysis to identify the best solutions with respect to fitness and spread that achieve optimal phenotypic trade-offs [82]. Similar Pareto-guided approaches have been successfully applied in other generative design domains, such as multi-objective molecular generation via Pareto-based reinforcement learning [83]. Genetic load estimates can be incorporated as penalty terms, and multi-context validation across environments, developmental stages, and genetic backgrounds should be embedded in the outer loop to uncover latent trade-offs. These outcomes should feed back into the generative model, enabling it to learn not only which designs succeed, but why.

## 6. Limitations, Risks, and Open Questions for Generative Plant Breeding

While the proposed framework offers a promising direction for generative plant design, its practical deployment faces several limitations and risks that must be critically examined. Below, we discuss key challenges across data, model, regulatory, and interpretability dimensions, followed by open questions for future research.

Data scarcity remains a fundamental bottleneck. Generative models require large-scale, high-quality training datasets to learn meaningful latent representations, yet collecting such data in plant breeding is constrained by cost, labor, and seasonality [84]. In barley growth analysis, the limited variability and scarcity of multivariate time-series data have motivated the use of VAEs to generate synthetic growth trajectories [85]. Similarly, a lesion information transfer diffusion model has been developed specifically to augment disease lesion datasets, improving diagnostic accuracy by more than 3% [86]. Transfer learning offers a complementary strategy. In peanut equivalent water thickness monitoring, a Wasserstein GAN was used to generate synthetic spectral data for pre-training, and the model was then fine-tuned with limited field data, achieving higher predictive accuracy than models trained on real data alone [87].

Model hallucinations pose a distinct risk in agricultural applications. In soybean cultivation, multimodal large language models have been shown to produce hallucinated recommendations with a rate as high as 54.17% when relying solely on text prompts [88]. In open-field white radish production, a hybrid large language model–quantitative model framework was developed specifically to reduce hallucinations in planting-to-harvesting decisions [89]. These examples demonstrate that hallucinations are a concrete obstacle to deploying generative AI in agricultural decision-making. This underscores the need for robust hallucination mitigation strategies before generative models can be reliably deployed in plant breeding. In plant breeding, hallucinations could manifest as genotypes that appear optimal in silico but carry hidden genetic load or pleiotropic trade-offs.

Uncertainty propagation compounds these risks. When generative models are trained or fine-tuned on their own outputs over successive rounds, errors can accumulate, leading to progressive drift from the target distribution [90]. This uncertainty further undermines reproducibility, which has become a critical concern in generative AI research [91]. Generative models are notoriously sensitive to hyperparameters and random seeds, making results difficult to replicate across studies.

Biosafety and regulatory challenges further complicate deployment. AI-generated genotypes may fall outside existing regulatory frameworks, particularly if they involve novel allele combinations or synthetic elements. In protein design, deep generative models can propose sequences with little or no homology to natural counterparts, potentially bypassing sequence-based biosafety checks [92] and references cited therein. In food enzyme engineering, AI-driven designs must be evaluated for allergenicity, toxicity, and genetic safety, yet current assessment methods face challenges in adapting to AI-generated sequences [93]. Current governance provides only partial coverage: the European Union AI Act does not regulate biological hazard assessments, World Health Organization instruments lack operational rules specific to AI-generated proteins, and the Cartagena Protocol, which regulates living modified organisms, does not address AI-designed protein sequences [92]. These governance gaps underscore the need for updated biosafety frameworks.

Finally, the interpretability of generated designs remains a persistent barrier. Generative models are largely “black boxes” that offer limited insight into the biological rationale behind their outputs. This opacity is not unique to plant breeding; in ecology, complex models have similarly been shown to limit scientific understanding and undermine trust in conservation decisions [94]. While efforts to build interpretability into generative models for plants are emerging, for example biomechanically guided frameworks that generate biologically plausible 3D maize structures from single images (MIRAGE) [95], such approaches remain nascent. More broadly, the application of explainable AI to plant breeding is still in early stages [96].

Despite recent progress, several fundamental open questions remain unresolved. These gaps become more urgent as extreme climate events intensify, calling for dedicated research to advance generative plant breeding toward practical deployment. First, the shift from static design targets to dynamic response programs is conceptually clear but technically underdeveloped. Latent space methods can in principle embed temporal trajectories, yet we still lack generative models that reliably synthesize regulatory architectures for homeorhesis. This gap is reflected in current AI-driven cis-regulatory element design, which remains predominantly confined to sequence-level features and has not yet achieved system-level regulatory network generation [97]. The design space for environmental sensing and feedback regulation far exceeds that for static design targets, and few generative models are optimized for control-oriented objectives.

Second, the growing gap between computational design and field performance remains an open question. Generative models trained on existing data often yield statistically plausible genotypes that fail in real environments due to hidden biological costs: cryptic deleterious mutations, unforeseen trait trade-offs, or incompatibility with local microbiomes. Trustworthy evaluation pipelines can reduce this cost by filtering out unreliable designs early. However, although DTs and surrogate models offer strategies to this goal, formal frameworks to estimate, predict, and minimize the alignment cost remain lacking.

Third, the field still lacks evaluation metrics that reflect biological function. The ultimate criterion should be causal efficacy: whether a designed genotype reliably produces the intended phenotypic effect under experimental conditions. Yet no standardized benchmarks exist that rank generative models based on their validation rates in perturbation tests. Initiatives such as CausalBench [98] provide a starting point for network inference, but extending this idea to generative plant breeding requires community-wide testbeds with well-characterized regulatory elements, genetic backgrounds, and high-throughput perturbation assays.

The integration of generative AI into plant breeding represents a move away from static genome optimization toward the engineering of dynamic, adaptive plant systems. Success will depend on closer integration between the inner and outer loops of the proposed framework, better function-first evaluation pipelines, and a breeding paradigm that sets more ambitious goals while remaining firmly grounded in biological constraints. If achieved, this transition could enable agricultural systems that are not only adapted to historical climates but also programmable to withstand future environmental uncertainty.

## Figures and Tables

**Table 1:** Comparison of predictive AI, generative AI, mechanistic models, and digital twins in the proposed generative design framework.

Model Type	Objective	Strength	Limitation	Data Requirement	Role in Framework
Predictive AI	Predict phenotypes from multi-omics or environmental inputs	Fast, scalable, well-established	Limited to training distribution, cannot generate novelty	Large labeled multi-omics datasets	Screens and evaluates candidate designs
Generative AI	Generate novel, plausible genotypes or designs	Enables de novo creation, explores unseen design space	May produce biologically implausible outputs, requires careful constraints	Large high-quality training datasets	Generates novel candidate designs
Mechanistic models	Simulate biological/physiological processes	Interpretable, capable of extrapolating beyond data	Require known mechanisms, computationally intensive	Mechanistic parameters and equations	Constrains candidate viability biophysically
Digital twins	Mirror and predict real-world plant/field behavior	Integrates data and mechanisms, real-time updating	Computationally expensive, requires mature mechanistic models for many traits	Real-time sensor data and process models	Filters candidates before field validation

## Data Availability

The data presented in this study (including the simulation results shown in Figure 4) were generated using the R code provided in Supplementary Code S1. No external datasets were used or analyzed.

## Supplementary Materials

The following supporting information can be downloaded at: https://www.mdpi.com/article/10.3390/plants15131967/s1, Supplementary Code S1: Simulation of the dual-loop generative design framework for drought-tolerant maize.
